# Presidential address: improving item validity and adopting computer-based testing, clinical skills assessments, artificial intelligence, and virtual reality in health professions licensing examinations in Korea

**DOI:** 10.3352/jeehp.2023.20.8

**Published:** 2023-03-27

**Authors:** Hyunjoo Pai

**Affiliations:** President, Korea Health Personnel Licensing Examination Institute, Seoul, Korea; Hallym University, Korea


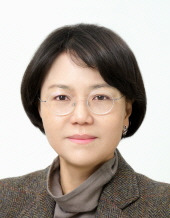


## Progress of health professions licensing examinations in Korea over 31 years

In 1992, the Korea Health Personnel Licensing Examination Institute (KHPLEI) was established to provide a national licensing examination system for healthcare professionals [1]. Since its inception, the KHPLEI has operated professionally, continuously researching and developing licensing examination systems. By ensuring the minimal competency of healthcare professionals through a stable licensing examination system, the KHPLEI has made significant contributions to the development of healthcare in Korea.

Over the past 3 decades, the KHPLEI has undergone significant developments through the dedicated efforts of its former directors, staff, and related organizations. Originally founded as an institute focusing on examinations for medical doctors, the KHPLEI has been restructured and expanded since 1998. As of 2023, the institution handles licensing examinations for 26 health professions. The institute also introduced clinical skill tests to the medical licensing examination in 2009, becoming the first in Asia to do so [2]. In addition, the KHPLEI established smart device-based testing [3] for the licensing examination of emergency medical technicians in 2017 and computer-based testing (CBT) [4] for the medical licensing examination in 2022.

## New development plan for health professions licensing examinations

As the 9th president of KHPLEI, I took office on February 1, 2023. With 30 years of experience as an infectious disease specialist and having taught many medical students and young doctors, I have deeply considered the mission, role, and necessary skills of healthcare professionals in the era of infectious disease epidemics during coronavirus disease 2019 pandemic. Based on my experience, I aim to manage the national licensing examinations for all healthcare professionals who can serve the Korean people and the global community. According to the institute’s goals, a new development plan for licensing examinations is suggested as follows:

First, we will reinforce the basics of licensing examinations by ensuring that the test items are valid for measuring examinees’ competencies and that the items are reliable. Developing items that effectively measure examinees’ deep knowledge and practical problem-solving abilities is the most fundamental task of the KHPLEI. To achieve this goal, we will prioritize close communication and comprehensive support from experts in each field and encourage multidisciplinary discussions and agreements to focus on intensive item development. Additionally, regular evaluations by experts will be intensified to review the reliability and validity of the test items.

Second, after introducing CBT to the medical licensing examinations in 2022, CBT was adopted for the licensing examinations of dentists, oriental medical doctors, emergency medical technicians, and care workers in 2023. The emergency medical technician licensing examination has been administered through smart device-based testing since 2017, but it was converted to CBT. In 2024, the examinations for midwives, oriental medicine pharmacists, health educators, and assistive technology professionals will be executed through CBT. We aim to expand CBT to all healthcare licensing examinations in the next few years to improve measurement quality and facilitate examinees’ ease in the testing environment. Beginning in 2023, the institute will administer the care worker licensing examination to 350,000 examinees annually as a year-round CBT. After analyzing the data from these examinations and experiences of testing, we will prepare for the implementation of computerized adaptive testing (CAT) for healthcare licensing examinations. Expert-suggested software [5], simulation studies [6,7], and item selection algorithms [8] will be utilized to ensure the successful implementation of CAT.

Third, we will enhance measurements of clinical competency. Despite the challenges posed by the pandemic, we have continued to conduct secure clinical skill tests for 6 professions, including physicians, emergency medicine technicians, dental technicians, dental hygienists, prosthetists/orthoptists, and dentists. We added a clinical skills examination for dental licensing in 2022 [1]. While accurate knowledge is essential for healthcare professionals, applying that knowledge in real-world situations and establishing good relationships with patients is equally important. Therefore, we aim to further develop clinical practice tests as a cornerstone of clinical practice education in medical/health training.

Fourth, we will diversify testing methods. Rapid advancements in technologies such as artificial intelligence (AI) and virtual reality (VR) are increasingly being applied daily. If AI can carry out tasks that currently take up much of a physician’s time, doctors can spend more time at the bedside [9]. VR medical simulations can provide various standardized learning experiences in medical education and testing, catering to different learning styles [10]. It is time to adopt these technologies to improve the quality of measurements of examinees’ competency. Although CBT has already provided video items, utilizing advanced technologies, including AI and VR, can help us measure a broader range of knowledge and clinical skills without space limitations. Research on adopting AI and VR for licensing examinations will be promoted.

In addition to these objectives, the institute has planned further initiatives. The KHPLEI signed a memorandum of understanding with the Vietnamese government in 2019 to transfer our expertise and has also cooperated with requests from other countries and institutions [11]. Likewise, we will continue to share our knowledge with other nations. For example, new specialized fields such as bioinformatics and digital therapeutic devices have recently gained attention in the medical industry. We aim to research and enhance the proficiency of examinees in these fields. As health professions that involve direct contact with patients require ethics, we intend to develop and implement an education and testing system to address medical ethics with more practical items. Several studies have been conducted on standard setting for paper-and-pencil testing or CBT to overcome the limitations of having the passing score set at 60% of the total score, which has been used since 1952 in health professions licensing examinations in Korea. The fixed passing score may cause variability in the proportion of passed examinees regardless of the examinees’ minimum requirements. The yes/no Angoff and Hofstee methods have been suggested for the Korean Medical Licensing Examination [12]. A revision of the law is required to adopt those standard-setting proposals, which is also an urgent task of KHPLEI.

## Continued support for official journal publishing

As the president of KHPLEI, I recognize the importance of disseminating valuable information to experts in licensing examinations and healthcare education. I also believe that support for publishing our official journal, *Journal of Educational Evaluation for Health Professions*, should be a core role of the KHPLEI. I will work closely with the editorial board of the journal and other stakeholders to develop strategies for sustaining and expanding the reach of the official journal.
